# Supplementary material for: Dynamics of Viral Shedding and Symptoms in Patients with Asymptomatic or Mild COVID-19
Source: Viruses. 2021 Oct 22;13(11):2133. doi: 10.3390/v13112133 (PMC8625453; doi:10.3390/v13112133)
Supplement: Supplementary file 1 [file viruses-13-02133-s001.zip › viruses-1387784-supplementary/viruses-1387784-si.pdf]

Supplementary Data – Questionnaire sheet for COVID-19 related symptoms

This is a questionnaire for monitoring daily symptoms of COVID-19 infection. To understand the dynamics of various symptoms after COVID-19 infection, we are going to collect and use personal information for monitoring all residents as follows. All information will be anonymized and fully encrypted before analysis. The survey collects residents' name and scoring of various symptoms.

◎ I agree to the collection and use of personal information.

① Yes ② No

(Question 1) Please give us some information about yourself.

1-1. Please fill in your name. ____________.

1-2. Please fill in today's date. .

(Question 2)

2-1. Do you have a fever above 37.5′C? ① Yes

2-2. Have you taken any antipyretics within 24 h? ① Yes

(Question 3) Symptom checklist with severity scores. You only need to check the items that you experienced. Unchecked items are considered symptom-free. Please score the symptoms as following criteria: score 0 (no symptom), score 1 (transient or mild discomfort, no interference with daily activity, and no requirement of medicine), score 2 (mild-to-moderate limitation in daily activity, and symptoms are controlled medicine) or score 3 (substantial limitation in daily activity and difficulty in control symptoms with medicine).

3-1. febrile sense ① 0 (none) ② 1 (mild) ③ 2 (moderate) ④ 3 (severe)

3-2. chilling sense ① 0 (none) ② 1 (mild) ③ 2 (moderate) ④ 3 (severe)

3-3. myalgia ① 0 (none) ② 1 (mild) ③ 2 (moderate) ④ 3 (severe)

3-4. fatigue ① 0 (none) ② 1 (mild) ③ 2 (moderate) ④ 3 (severe)

3-5. headache ① 0 (none) ② 1 (mild) ③ 2 (moderate) ④ 3 (severe)

3-6. dizziness ① 0 (none) ② 1 (mild) ③ 2 (moderate) ④ 3 (severe)

3-7. skin rash ① 0 (none) ② 1 (mild) ③ 2 (moderate) ④ 3 (severe)

3-8. conjunctival hemorrhage ① 0 (none) ② 1 (mild) ③ 2 (moderate) ④ 3 (severe)

3-9. cough ① 0 (none) ② 1 (mild) ③ 2 (moderate) ④ 3 (severe)

3-10. sputum ① 0 (none) ② 1 (mild) ③ 2 (moderate) ④ 3 (severe)

3-11. sore throat ① 0 (none) ② 1 (mild) ③ 2 (moderate) ④ 3 (severe)

3-12. runny nose ① 0 (none) ② 1 (mild) ③ 2 (moderate) ④ 3 (severe)

3-13. stuffy nose ① 0 (none) ② 1 (mild) ③ 2 (moderate) ④ 3 (severe)

3-14. dyspnea ① 0 (none) ② 1 (mild) ③ 2 (moderate) ④ 3 (severe)

3-15. chest pain ① 0 (none) ② 1 (mild) ③ 2 (moderate) ④ 3 (severe)

3-16. taste disorder ① 0 (none) ② 1 (mild) ③ 2 (moderate) ④ 3 (severe)

3-17. olfactory disorder ① 0 (none) ② 1 (mild) ③ 2 (moderate) ④ 3 (severe)

3-18. nausea ① 0 (none) ② 1 (mild) ③ 2 (moderate) ④ 3 (severe)

3-19. anorexia ① 0 (none) ② 1 (mild) ③ 2 (moderate) ④ 3 (severe)

3-20. dyspepsia ① 0 (none) ② 1 (mild) ③ 2 (moderate) ④ 3 (severe)

3-21. vomiting ① 0 (none) ② 1 (mild) ③ 2 (moderate) ④ 3 (severe)

3-22. diarrhea ① 0 (none) ② 1 (mild) ③ 2 (moderate) ④ 3 (severe)

3-23. constipation ① 0 (none) ② 1 (mild) ③ 2 (moderate) ④ 3 (severe)

3-24. abdominal pain ① 0 (none) ② 1 (mild) ③ 2 (moderate) ④ 3 (severe)

Measurement of viral load by real-time RT-PCR assay

Multiplex RT-PCR assay mix (20 μL) contained 4 μL of 5X master mix (LightCycler Multiplex RNA Virus Master, Roche, Basel, Switzerland), 0.1 μL of 200X enzyme mix, 500 nM of each S and N gene primer, 200 nM of each S and N gene probe, 250 nM of internal control primers, 100 nM of internal control probes, and 5 μL of extracted RNA or in vitro-synthesized control RNA. PCR amplification was performed with a LightCycler 96 system (Roche) in the following conditions: reverse transcription at 50℃ for 10 min, initial denaturation at 95℃ for 5 min, 45 cycles of 2-step amplification, denaturation at 95℃ for 10 s, and final extension at 60℃ for 30 s. To generate calibration curves, serial dilutions from 10^7 to 5 copies/μL of synthetic control RNA were assayed in six independent sets of reactions (Supplementary Figure 4). The detection limit of this assay was 5 copies/reaction (2.6 log copies/mL of specimen) and viral copy numbers were determined by plotting CT values against log copies/reaction.

Detection of N and S gene subgenomic RNAs

Multiplex real-time RT-PCR assay reaction mixture and cycling condition were same as the condition of the RT-PCR for viral loads measurement described in the Supplemental Materials as above. The result was considered as a positive if the cycle threshold values of both N and S subgenomic RNAs were less than 36. The limit of detection of this assay was 5 copies/reaction.

Table S1. Primers and probes used for real-time RT-PCR assay for the detection of N gene and S gene of SARS-CoV-2

Target (Accession #)	Name	Location	Sequence	Modification
N gene (NC_045512)	NF	29356	AACATTCCCACCAACAGAGC	
	NR	29529	GCCTGAGTTGAGTCAGCACT	
	NP	29462	GCTGATGAAACTCAAGCCTTACCGCA	5′Cy5, 3′BHQ2
S gene (NC_045512)	SF	21624	GAACTCAATTACCCCCTGCAT	
	SR	21787	ACCATTGGTCCCAGAGACAT	
	SP	21657	TCACACGTGGTGTTTATTACCCTGACA	5′FAM, 3′BHQ1
Internal control (NC_000007.14)	BAF	1670	ACTAACACTGGCTCGTGTGA	
	BAR	1774	CTTGGGATGGGGAGTCTGTT	
	BAP	1700	AGGCTGGTGTAAAGCGGCCTTGG	5′HEX, 3′BHQ1

Footnote. The samples were considered positive for SARS-CoV-2 genomic RNA detection if both N and S genes were positive as well as the positive internal control.

Table S2. Primers and probes used for the detection of N gene and S gene of subgenomic RNAs of SARS-CoV-2

Target*	Name	Location	Sequence	Modification
5′ leader	SG-F	15	CCTTCCCAGGTAACAAACCA	
N gene	SGRT-NR2	28436	CGGTGAACCAAGACGCAGTA	
	SGRT-NP2	28322	TTTGGTGGACCCTCAGATTCAACTGG	5′-FAM, 3′-BHQ1
S gene	SGRT-SR1	21678	GGGTAATAAACACCACGTGTGAA	
	SGRT-SP1	21617	ACAACCAGAACTCAATTACCCCCTGCA	5′-CY5, 3′-BHQ2

(*Accession #: NC_045512.2)

Table S3. Frequency and duration of COVID-19-related symptoms in symptomatic and presymptomatic patients

	**Symptom during admission, n (%)**				**Initial symptom, n (%)**			**Symptom duration, median (IQR)**		
	**Total (n=77)**	**Symptomatic (n=61)**	**Presymptomatic (n=16)**	***P* value**	**Symptomatic (n=61)**	**Presymptomatic (n=16)**	***P* value**	**Symptomatic (n=61)**	**Presymptomatic (n=16)**	***P* value**
Febrile sense	47 (61)	41 (62)	6 (38)	.09	32 (52)	5 (31)	.17	2 (1–4)	3 (1–6)	.91
Chills	28 (36)	25 (38)	3 (19)	.24	17 (28)	1 (6)	.10	3 (1–6)	9 (1–9)	.25
Myalgia	38 (49)	34 (52)	4 (25)	.09	25 (41)	2 (13)	.041	5 (1.75–8.25)	6 (2.25–8.25)	.93
Fatigue	47 (61)	38 (58)	9 (56)	>.99	29 (48)	6 (38)	.58	6 (3–10)	5 (1–10)	.36
Headache	44 (57)	36 (55)	8 (50)	.79	29 (48)	3 (19)	.048	6 (2–8.75)	3 (1.25–7.75)	.42
Dizziness	24 (31)	21 (32)	3 (19)	.37	18 (30)	1 (6)	.10	2 (1–8)	10 (2–11)	.10
Cough	56 (73)	45 (68)	11 (69)	.57	34 (56)	8 (50)	.78	9.5 (6–11)	7.5 (4–9.75)	.11
Sputum	55 (71)	45 (68)	10 (63)	.77	34 (56)	8 (50)	.78	10 (6.5–11)	5 (1–9.5)	.04
Sore throat	42 (55)	36 (55)	6 (38)	.27	30 (49)	5 (31)	.26	6 (3–9)	4.5 (1–9.25)	.50
Rhinorrhea	43 (56)	39 (59)	4 (25)	.02	20 (33)	3 (19)	.37	6 (3–9)	6.5 (2.25–8.5)	.69
Nasal congestion	45 (58)	39 (59)	6 (38)	.16	17 (28)	4 (25)	>.99	8 (5–10)	3.5 (2–7.25)	.07
Dyspnea	21 (27)	18 (27)	3 (19)	.75	12 (20)	3 (19)	>.99	5 (1–7)	1 (1–9)	.64
Chest pain	14 (18)	13 (20)	1 (6)	.28	7 (11)	2 (13)	>.99	2 (1–6)	1.5 (1–2)	.57
Taste disorder	42 (55)	35 (53)	7 (44)	.58	14 (23)	4 (25)	>.99	9 (6–11)	8 (5–11)	.81
Olfactory disorder	43 (56)	37 (56)	6 (38)	.27	13 (21)	1 (6)	.28	10 (7.5–11)	11 (9.5–11)	.18
Anorexia	47 (61)	39 (59)	8 (50)	.58	25 (41)	5 (31)	.57	9 (4–11)	10 (2.25–11)	.79
Dyspepsia	24 (31)	22 (33)	2 (13)	.13	12 (20)	1 (6)	.28	4 (2–9)	2.5 (1–4)	.41
Abdominal pain	14 (18)	12 (18)	2 (13)	.73	6 (10)	0 (0)	.33	2 (1–4.75)	6.5 (3–10)	.34
Nausea	15 (19)	11 (17)	4 (25)	.48	7 (11)	0 (0)	.33	2 (1–7)	8 (5–10.25)	.09

Vomiting	6 (8)	4 (6)	2 (13)	.33	0 (0)	0 (0)	NA	9 (6.75–9.75)	4.5 (4–5)	.07
Diarrhea	22 (29)	18 (27)	4 (25)	>.99	9 (15)	2 (13)	>.99	5.5 (2.75–8)	3 (1.5–4.5)	.18
Constipation	17 (22)	15 (23)	2 (13)	.50	6 (10)	0 (0)	.33	6 (4–6)	3.5 (2–5)	.33
Conjunctival hemorrhage	8 (10)	6 (9)	2 (13)	.65	4 (7)	2 (13)	.60	3.5 (1–6.5)	1 (1–1)	.36
Skin rash	5 (6)	5 (8)	0 (0)	.58	2 (3)	0 (0)	>.99	8 (3–10.5)	0 (0–0)	NA

NA, not available

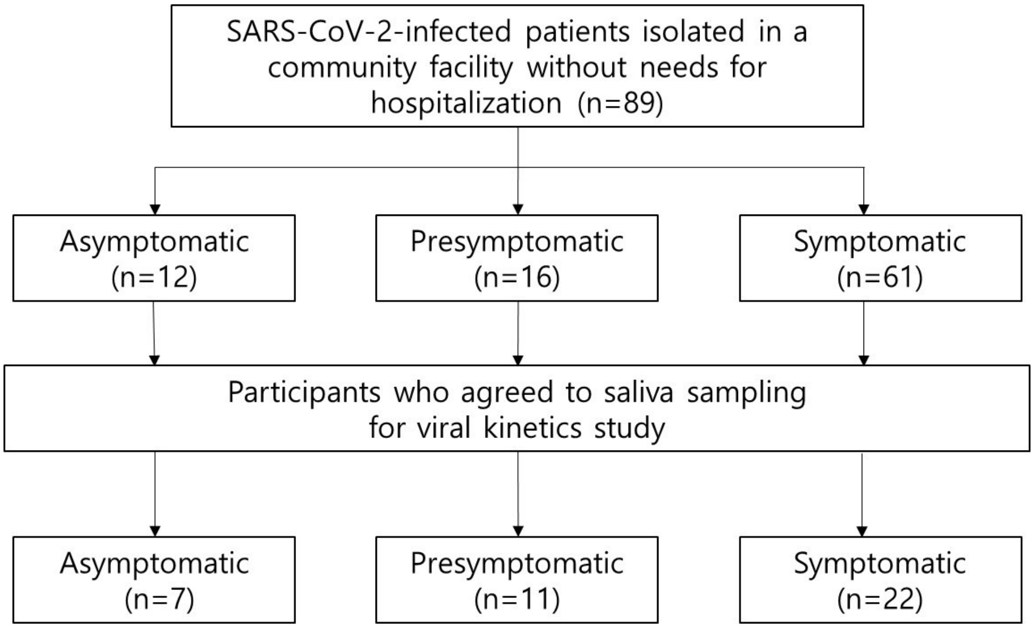

Figure S1. Flowchart of study participants

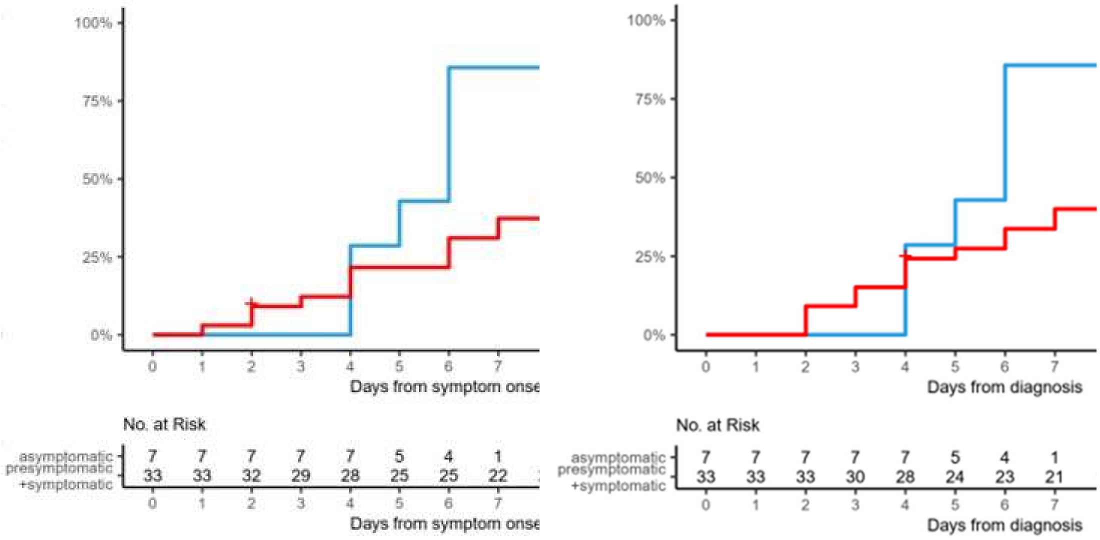

(**a**) (**b**)

Figure S2. Supplementary Figure 2. Kaplan-Meier curves for negative conversion proportion between symptomatic (including presymptomatic) and asymptomatic patients. *A*. Days from symptom onset. *B*. Days from diagnosis

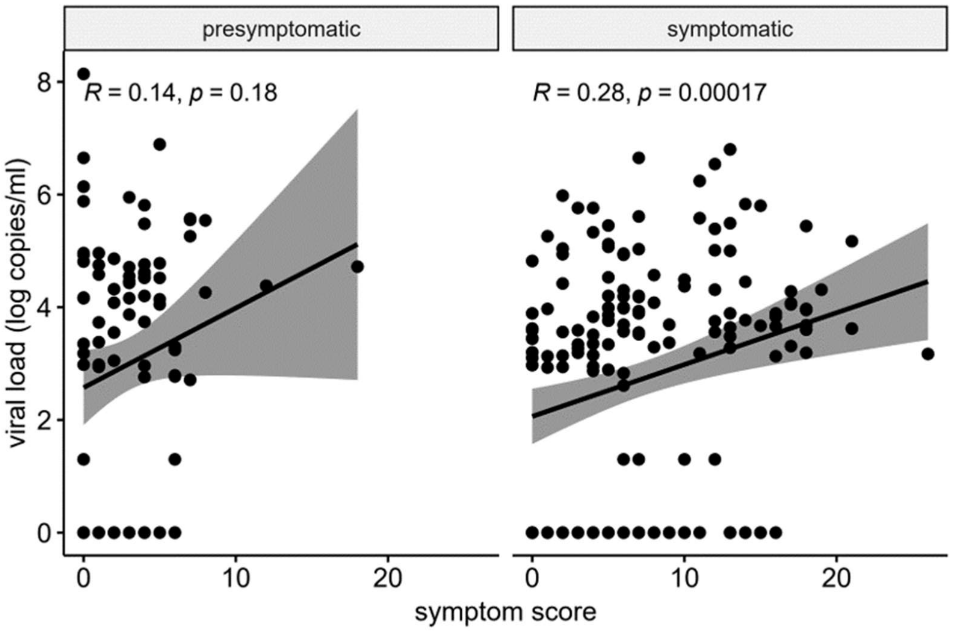

Figure S3. Correlation analysis between symptom score and viral load. Black line indicates linear regression line, and grey shadow indicates the 95% confidence interval.

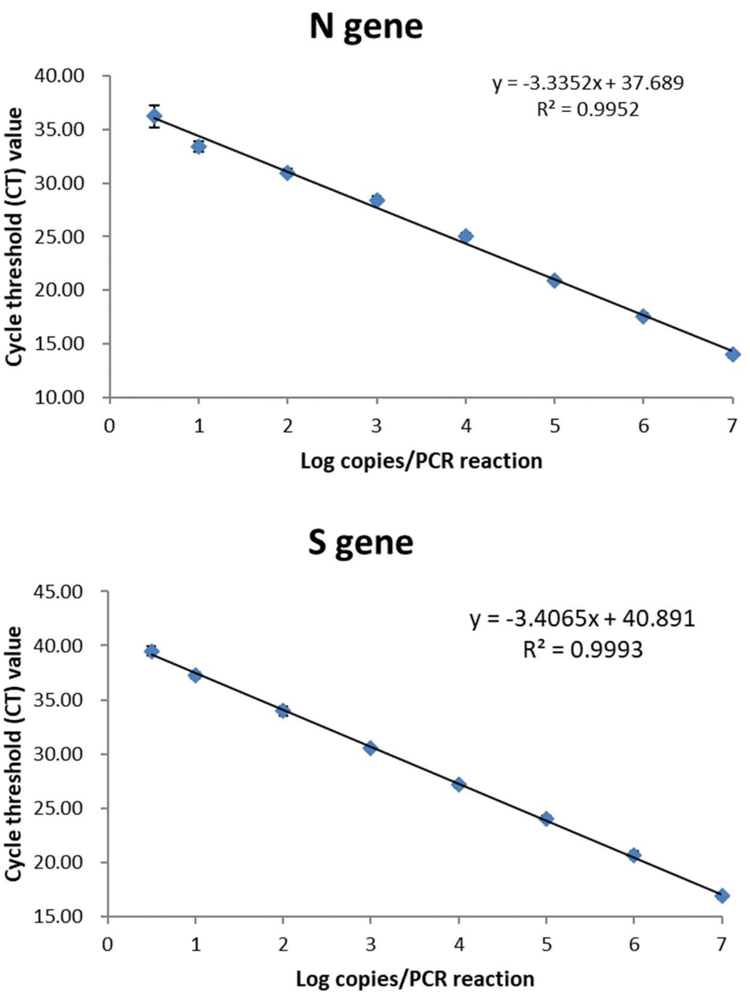

Figure S4. Correlation curves for cycle thresholds versus copies of the N and S genes
